# Blockade of senescence‐associated microRNA‐195 in aged skeletal muscle cells facilitates reprogramming to produce induced pluripotent stem cells.

**DOI:** 10.1111/acel.12411

**Published:** 2015-12-05

**Authors:** Hideyuki Kondo, Ha Won Kim, Lei Wang, Motoi Okada, Christian Paul, Ronald W. Millard, Yigang Wang

**Affiliations:** ^1^ Department of Pathology and Lab MedicineUniversity of Cincinnati231 Albert Sabin WayCincinnatiOH45267USA; ^2^ Department of Internal MedicineVascular Biology CenterMedical College of GeorgiaGeorgia Regents University1429 Laney Walker BlvdAugustaGA30912USA; ^3^ CardiovascularDivision of Coronary Heart DiseaseDepartment of Internal MedicineHyogo College of Medicine1‐1 MukogawaNishinomiyaHyogo663‐8131Japan

**Keywords:** aging cell, microRNA, reprogramming, telomere length

## Abstract

The low reprogramming efficiency in cells from elderly patients is a challenge that must be overcome. Recently, it has been reported that senescence‐associated microRNA (miR)‐195 targets Sirtuin 1 (SIRT1) to advance cellular senescence. Thus, we hypothesized that a blockade of miR‐195 expression could improve reprogramming efficiency in old skeletal myoblasts (SkMs). We found that miR‐195 expression was significantly higher in old SkMs (24 months) isolated from C57BL/6 mice as compared to young SkMs (2 months, 2.3‐fold). Expression of SIRT1 and telomerase reverse transcriptase (TERT) was downregulated in old SkMs, and transduction of old SkMs with lentiviral miR‐195 inhibitor significantly restored their expression. Furthermore, quantitative *in situ* hybridization analysis demonstrated significant telomere elongation in old SkMs transduced with anti‐miR‐195 (1.7‐fold increase). It is important to note that blocking miR‐195 expression markedly increased the reprogramming efficiency of old SkMs as compared to scramble (2.2‐fold increase). Transduction of anti‐miR‐195 did not alter karyotype or pluripotency marker expression. Induced pluripotent stem cells (iPSCs) from old SkMs transduced with anti‐miR‐195 successfully formed embryoid bodies that spontaneously differentiated into three germ layers, indicating that deletion of miR‐195 does not affect pluripotency in transformed SkMs. In conclusion, this study provided novel evidence that the blockade of age‐induced miR‐195 is a promising approach for efficient iPSC generation from aging donor subjects, which has the potential for autologous transplantation of iPSCs in elderly patients.

## Introduction

Current statistics indicate that a greater portion of the worldwide population is considered of advanced age (e.g., 25% in Japan, 21% in Germany, 17% in U.K., and 14% in U.S. populations are age 65 and older in 2013) according to a report released by World Population Review or NationMaster (http://www.nationmaster.com/). The demand for curative therapies for age‐related intractable and progressively degenerative diseases and disorders such as Alzheimer's, atherosclerosis, heart failure, liver cirrhosis, Parkinson's, and renal failure is at an all‐time high among elderly patients. The use of embryonic stem cells (ESCs) affords a feasible approach to regenerating tissue as a redress for degenerated organs and has offered some hope to elderly patients suffering from such degenerative disorders (Evans & Kaufman, 1981). In 2006, Yamanaka's research group in Japan reported that somatic cells could be reprogrammed by the expression of four factors associated with pluripotency, the so‐called Yamanaka factors: OCT4, SOX2, KLF4, and c‐Myc (OKSM) (Takahashi & Yamanaka, 2006). The emergence of induced pluripotent stem cell (iPSCs) technology has resulted in a paradigm shift in stem cell biology not only for regenerative medical techniques, but also for disease modeling and drug screening in relevant cellular systems for patients with degenerative diseases. Unlike ESCs, iPSCs are not subject to immune rejection because they are derived from a patient's own cells (Isobe *et al*., 2014), and present no ethical harvesting concerns related to the obtaining human ESCs for derivation. Although murine studies reveal that cell reprogramming efficiency declines with age, the potential to generate iPSCs from a patient's own cells has emerged as a promising strategy for autologous cell‐based therapy (Li *et al*., 2009; Kim *et al*., 2010; Cheng *et al*., 2011; Wang *et al*., 2011; Rohani *et al*., 2014).

MicroRNAs (miRs) are a class of noncoding RNAs that have been shown to play a significant role in gene regulation by targeting a variety of transcripts via a short region of imperfect complementarity termed the ‘seed region’. miRs have been identified recently as important regulators of cellular senescence and aging (Smith‐Vikos & Slack, 2012). Specifically, several miRs have been shown to regulate the Sirtuin 1 (SIRT1) signaling pathway. SIRT1 is known as a highly conserved NAD^+^‐dependent deacetylase, belonging to class III histone/protein deacetylases and is a member of the silent information regulator (Sir2) family (Longo & Kennedy, 2006). Furthermore, SIRT1 protein is highly expressed in ESCs, and transient overexpression enhances efficiency of the generation of iPSCs derived from mouse embryonic fibroblasts through miR‐34a‐SIRT1‐p53 pathways (Lee *et al*., 2012). Consistent with previous reports, our computational analysis using online software (TargetScan is copyrighted by the Whitehead Institution of Biomedical Research Compatibility) identified the predicted targets of miR‐195 as SIRT1 and telomerase reverse transcriptase (TERT) (Zhu *et al*., 2011).

Telomeres are special structures at the end of chromosomes that contain long tandem repeats of the DNA sequence TTAGGG, and the reduction in telomere length results in genetic instability with the passage of time. Conversely, telomere elongation mediated by TERT balances the progressive shortening of telomere length. Interestingly, reprogrammed cells regain pluripotency, previously considered irreversibly lost after a finite number of cell divisions. Moreover, endogenous TERT expression is induced with telomere elongation in the reprogramming process. TERT‐deficient mice exhibit a sharp reduction in reprogramming efficiency that can be restored by the reintroduction of TERT (Kinoshita *et al*., 2014).

Here, we investigated the influence of age on the character and reprogramming efficiency of primary skeletal myoblasts (SkMs). Furthermore, we set out to determine the effects produced by the inhibition of miR‐195 combined with the classic Yamanaka factors on reprogramming old cells into iPSCs.

## Results

### Expression of miR‐195 and aging markers is increased in old SkMs

**Figure 1:**
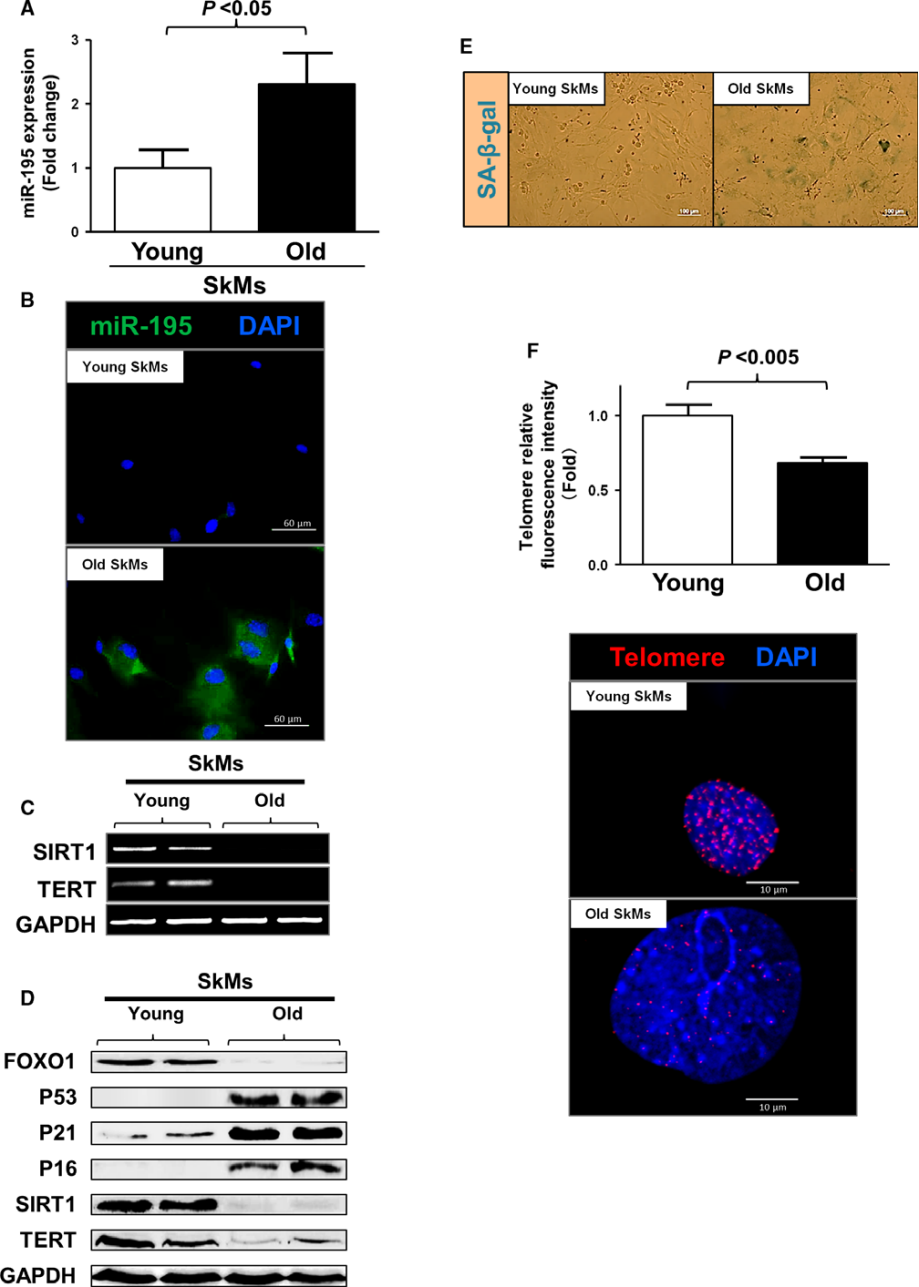
Expression of miR‐195 and aging markers increased in old SkMs. (A) Primary SkMs were cultured from 7 young and 7 old mice. miR were extracted from each primary cultured SkMs without passage. Higher miR‐195 expression was observed in cultured aged SkMs as shown by real‐time PCR (*P* < 0.05 vs. cultured young SkMs). (B) Expression of miR‐195 in aged SkMs was confirmed by FISH. (C, D) Primary SkMs were extracted from 4 young and 4 old mice. The old SkMs showed higher expression of senescence‐associated markers and lower expression of anti‐aging markers as examined by RT–PCR and Western blot. (E) SkMs isolated from old mice showed large number of SA‐β‐gal‐positive cells after the fifth passages. No positive cells were observed in cultured young SkMs even in the fifth passages. (F) The integrated red fluorescence intensity value for each telomere, which is proportional to the number of hybridized probes, was calculated and presented. Their average fluorescence intensity on cultured SkMs on the slides was higher in young SkMs (*P* < 0.005 vs. old SkMs).

MiR‐195 levels were measured in cultured SkMs without passage taken from both young and old mice (Fig. 1A). Quantitative RT–PCR analysis showed significantly higher levels of miR‐195 in old SkMs compared with young ones (2.3‐fold higher in old SkMs, *P* < 0.05). The same pattern was also observed using fluorescent in situ hybridization (FISH) analysis of green colored signal (Fig. 1B). Interestingly, there was negligible expression of miR‐195 in young SkMs, whereas old SkMs showed significantly higher miR‐195 expression as examined by FISH (green). The cellular phenotype between SkMs from young and old mice was next compared to elucidate the underlying mechanisms involved with miR‐195 in SkMs derived from old mice. RNA and protein samples were isolated from cultured cells without passage. RT–PCR data showed the mRNA levels of Sirt1 and Tert (targets of miR‐195 known as senescence‐related genes in old SkMs) were downregulated (Figs 1C and S1). Western blot analysis revealed diminished protein levels of SIRT1 and TERT as well as forkhead box transcription factor O1 (FOXO1) that interacts closely with SIRT1 (Figs 1D and S2A,E,F) (Xiong *et al*., 2011). As there is evidence that senescent cells can accumulate in renewable tissues with age (Herbig *et al*., 2006), the expression levels of proteins, including p53, p21, and p16, were next evaluated by Western blot to analyze the activation of senescence pathways in SkMs. Protein levels linked to senescence pathways (i.e., P53, P21, and P16) were upregulated in SkMs obtained from old mice (Figs 1D and S2B,C,D). We also compared senescence‐associated (SA) β‐gal expression in SkMs from young mice with those of old mice and observed that expression of SA‐β‐gal‐positive cells was markedly higher in old SkMs after the fifth passage (Fig. 1E).

As telomere shortening is associated with aging, telomere length was compared between young and old SkMs using quantitative FISH analysis. The histogram analysis based on fluorescence intensity revealed that old mice have shorter telomere length as compared to those taken from young mice as shown in Fig. 1F (old SkMs have 70% of fluorescence intensity compared to that of young ones, *P* < 0.005).

### Inhibition of age‐induced miR‐195 reverses aging marker expression

**Figure 2:**
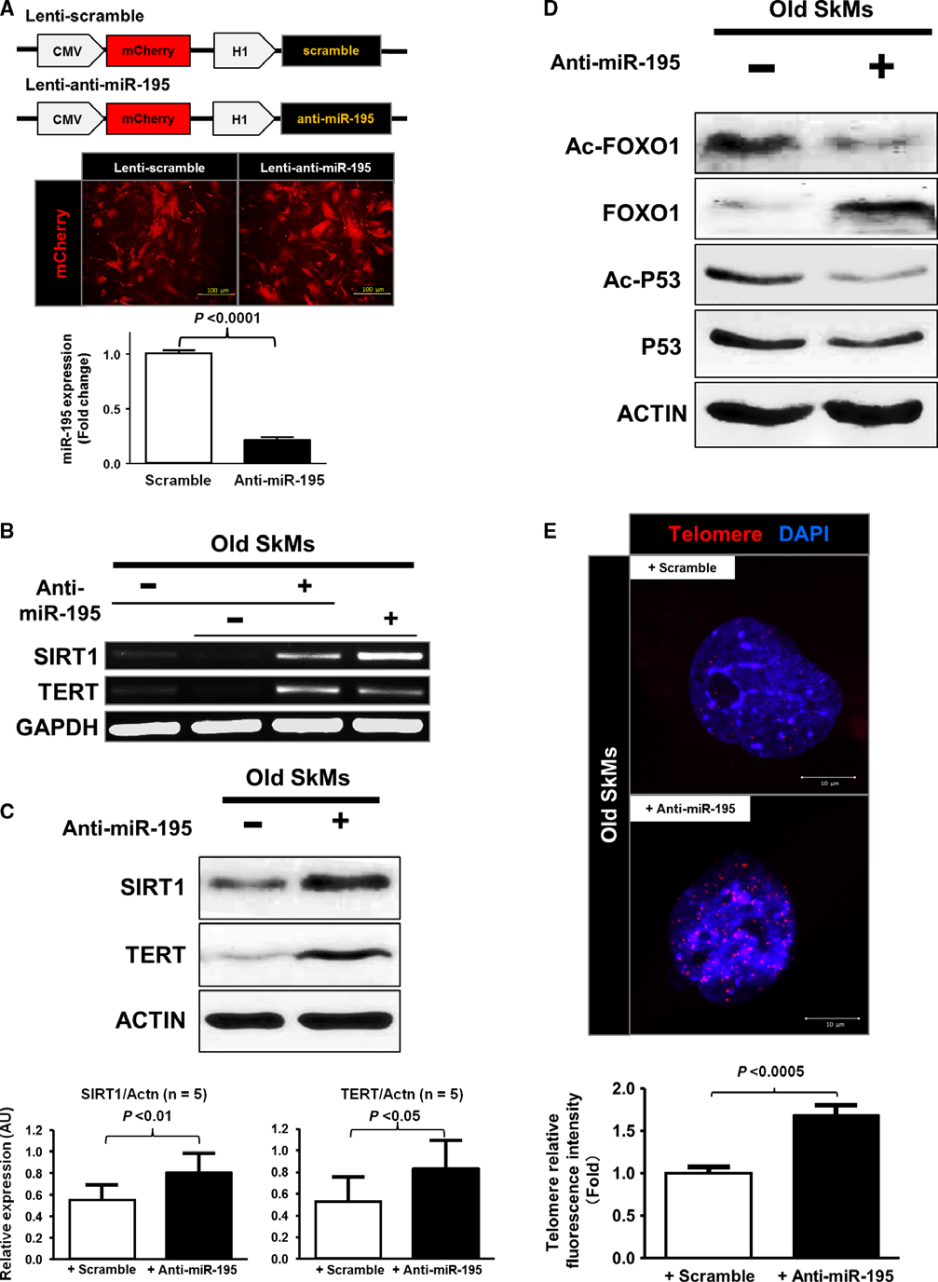
Inhibition of age‐induced miR‐195 reverses expression of senescence‐associated markers. (A) Transduction of lenti‐scramble or lenti‐miR‐195 inhibitor was monitored by mCherry reporter expression as red fluorescence and confirmed by qRT–PCR. (B, C) Transduction of old SkMs with miR‐195 inhibitor significantly restored mRNA expression of Sirt1 and Tert. Similar pattern was observed in old SkMs with miR‐195 inhibitor, restoring both SIRT1 (*P* < 0.01 vs. with scramble control) and TERT expressions (*P* < 0.05 vs. with scramble control). (D) Transduction of miR‐195 inhibitor altered the expression levels of age‐associated proteins such as FOXO1 and P53. Downregulation of acetylated proteins in FOXO1 and P53 was observed in cultured old SkMs with miR‐195, suggesting involvement of deacetylase like SIRT1. (E) The integrated red fluorescence intensity value for each telomere was calculated and presented. Their average fluorescence intensity on cultured SkMs on the slides was higher in old SkMs with miR‐195 inhibitor (*n *= 10 nuclei each, *P* < 0.0005 vs. old SkMs with scramble control).

The effects of miR‐195 inhibition were first examined against the already‐activated aging pathway in old SkMs to gain a better understand of its role. Using a lentiviral vector (lenti‐anti‐miR‐195) that could be successfully transduced in old SkMs, blockade of miR‐195 was achieved (4.5‐fold reduction was observed in old SkMs with anti‐miR‐195 assessed by qRT–PCR, *n *= 4, *P* < 0.0001) and mCherry signal (red fluorescence) in this vector system allowed us to determine transduction efficiency of lenti‐anti‐miR‐195 or scramble vector (Fig. 2A). TERT is a key protein in telomerase and, by controlling telomere length, serves a critical component in telomere replication and stabilization. Inhibition of miR‐195 was sufficient to release target genes Sirt1 and Tert (Figs 2B and S3A,B). Western blot analyses showed that miR‐195 inhibitor significantly restored the protein levels of both SIRT1 and TERT as shown in Figs 2C and S4 (1.5‐fold higher in SIRT1 levels with anti‐miR‐195 and 1.6‐fold higher in TERT levels with anti‐miR‐195 compared to control group, *P* < 0.01, *P* < 0.05, respectively). Inhibition of miR‐195 decreased acetylation of both FOXO1 and p53 (Fig. 2D). The effect of miR‐195 inhibition in telomere length was assessed by determining the upregulation of TERT by miR‐195 inhibitor in old SkMs. FISH analysis showed the telomere elongation by miR‐195 inhibitor in old SkMs as shown in Fig. 2E (anti‐miR‐195 group has 170% of fluorescence intensity compared to that of control group, *P* < 0.0005).

### Inhibition of age‐induced miR‐195 increases reprogramming efficiency in old SkMs

**Figure 3:**
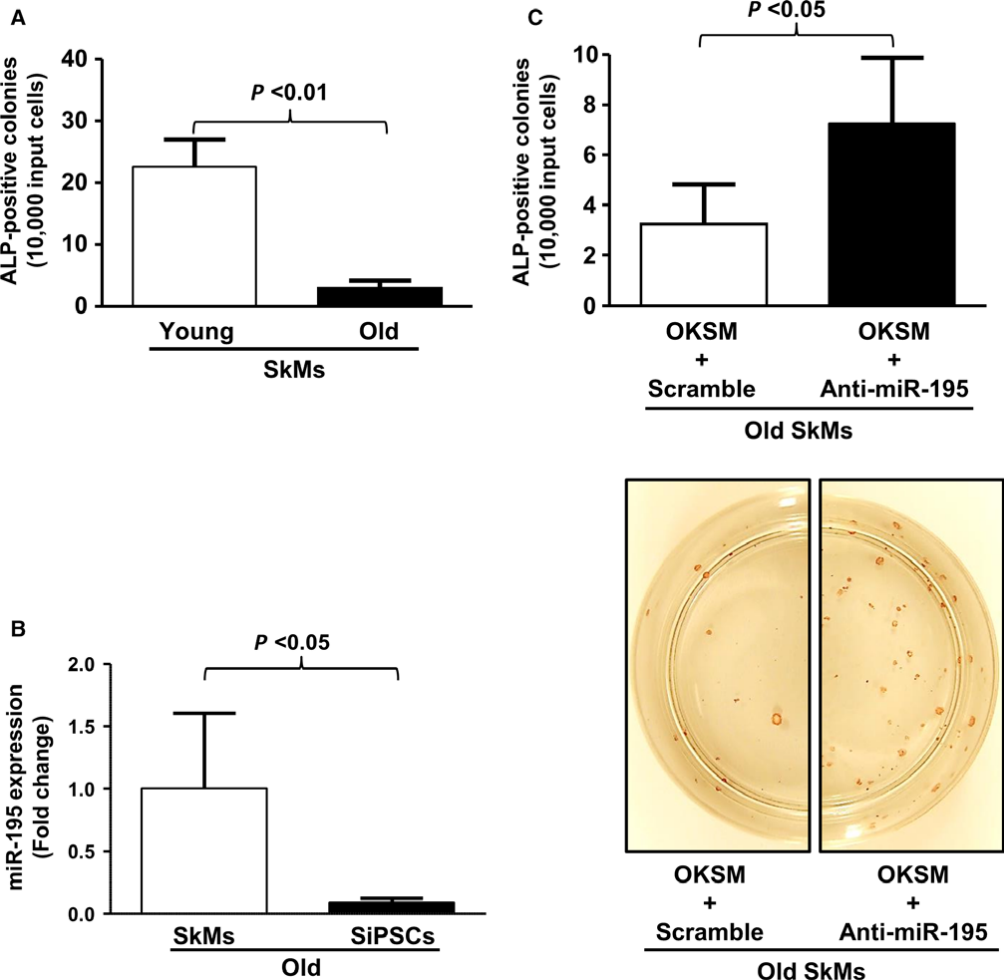
Inhibition of age‐induced miR‐195 increases reprogramming efficiency in old SkMs. (A) SkMs from old mice showed less efficient reprogramming efficiency for iPSCs generation as compared to those of young mice (young: 22.5 ± 4.6 vs. old: 3.6 ± 1.4 colonies per 1 × 10^4^ SkMs, *n *= 5 mice each, *P* < 0.01). (B) The expression level of miR‐195 was significantly reduced in iPSCs as compared to parental SkMs (*n *= 4 mice, *P* < 0.05). (C) Transduction of miR‐195 inhibitor together with OKSM improved the reprogramming efficiency of *de novo* iPSCs generation (scramble: 3.3 ± 1.6 vs. anti‐miR‐195: 7.3 ± 2.6 colonies per 1 × 10^4^ SkMs, *n *= 6 mice each, *P* < 0.05). Bars represent the number of ALP ^+^ colonies/10 000 cells plated initially.

Recently, aging has emerged as one of the leading factors associated with low reprogramming efficiency for *de novo* iPSCs generation, and we also observed similar reprogramming decreases in SkMs from old mice (Fig. 3A) as reported in earlier findings (Li *et al*., 2009; Kim *et al*., 2010; Cheng *et al*., 2011; Wang *et al*., 2011; Rohani *et al*., 2014). We reprogrammed SkMs from both young and old mice using classic Yamanaka factors, and colonies were stained with alkaline phosphatase (ALP) on day 16–18 and counted to assess reprogramming efficiency. This set of reprogramming experiments was repeated several times using primary cultured SkMs from a single mouse in each group, and 2 plates of 6‐cm dish were prepared in each experimental set. Significantly lower reprogramming efficiency was found in old SkMs compared with young SkMs (see Fig. 3: young: 22.5 ± 4.6 vs. old: 3.6 ± 1.4 colonies per 1 × 10^4^ SkMs, *n *= 5 mice each, *P* < 0.01). The findings in Figs 1 and 2 suggest that miR‐195 plays a key role in the modulation of senescence‐related signaling pathways, and we hypothesized that changes in the expression of miR‐195 could increase barriers to reprogramming cells derived from older subjects. Additional SkMs were prepared from 4 old mice and 2 iPSC colonies were chosen in each set for reprogramming. Real‐time PCR was performed in both old SkMs and iPSCs derived from the SkMs (SiPSCs) and showed significantly downregulated expression of miR‐195 in SiPSCs (Fig. 3B). Endogenous miR‐195 expression in SiPSCs had 11‐fold lower levels of miR‐195 in old SkMs (*n *= 4 mice, *P* < 0.05). A similar trend was also observed in young SkMs and the SiPSCs (*n *= 4 mice, *P* < 0.01) (Fig. S5). Old SkMs were then reprogrammed with the four classic Yamanaka factors together with miR‐195 inhibitor or the scramble control vector. Colony numbers were markedly increased under the condition that Yamanaka factors were combined with miR‐195 inhibitor, as examined by ALP staining on days 16–18 (Fig. 3C, indicating that the inhibition of miR‐195 significantly improved reprogramming efficiency in old SkMs (scramble: 3.3 ± 1.6 vs. anti‐miR‐195: 7.3 ± 2.6 colonies per 1 × 10^4^ SkMs, *n *= 6 mice each, *P* < 0.05)). However, the addition of the miR‐195 inhibitor together with OKSM did not affect the reprogramming efficiency in young SkMs (Fig. S6).

### Characterization and functional analysis of *de novo* iPSCs derived from SkMs using classic Yamanaka factors with miR‐195 inhibitor

**Figure 4:**
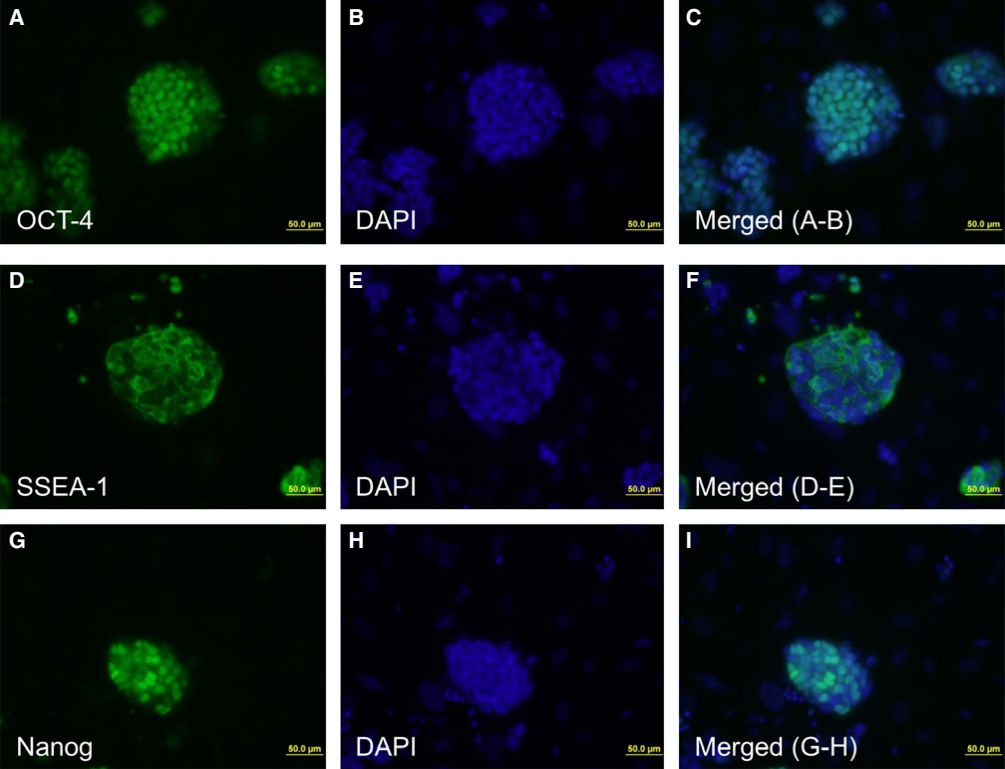
Characterization of iPSCs produced from old SkMs with miR‐195 inhibitor. (A–I) Immunostaining patterns for Oct4, SSEA‐1, and Nanog. C is a merged image from A and B. F is a merged image from D and E. I is a merged image from G and H. Nanog expression was not homogeneous in the iPSCs as commonly known.

**Figure 5:**
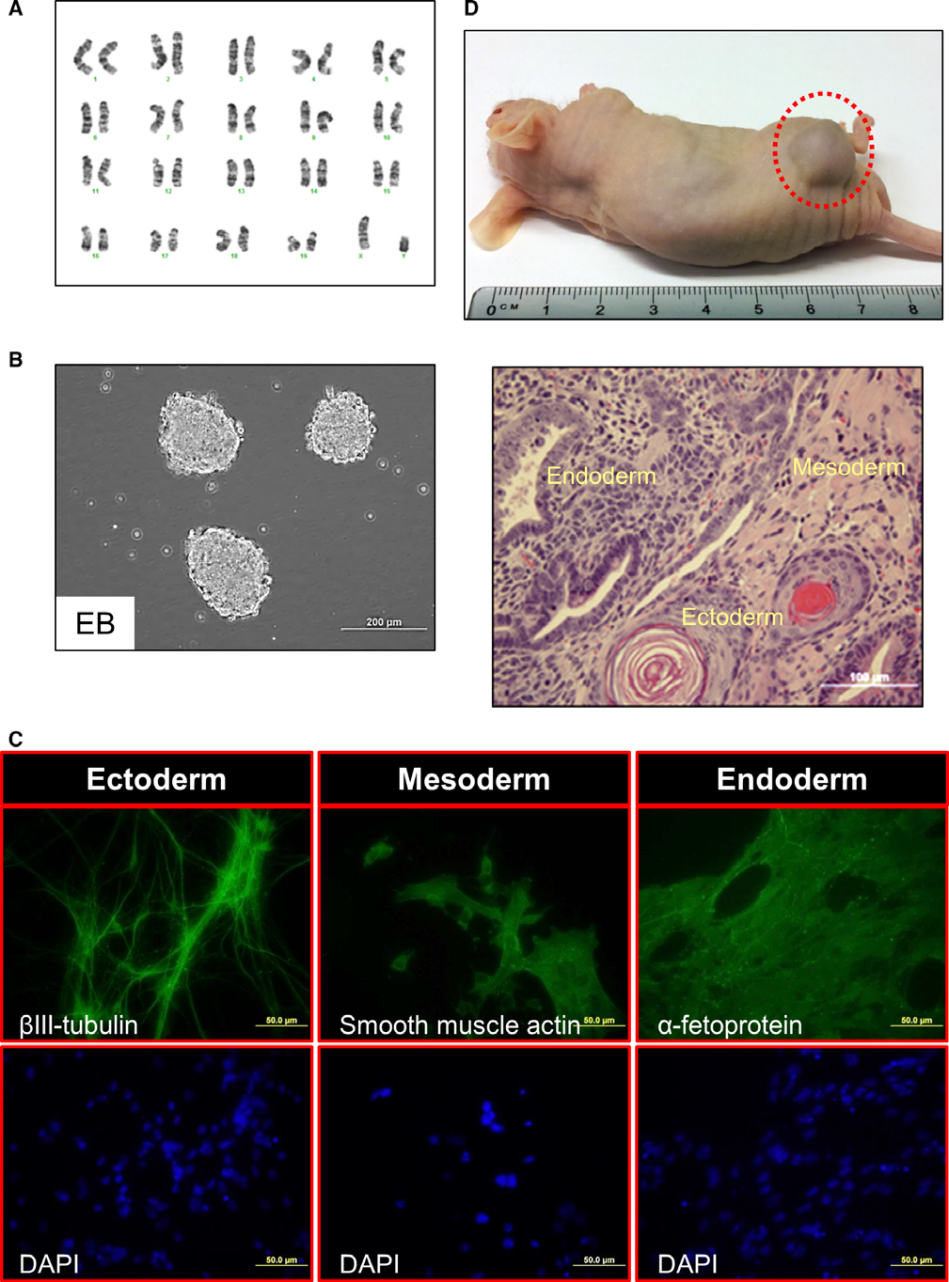
Functional analysis of iPSCs generated from old SkMs with miR‐195 inhibitor. (A) Karyotype was not altered in anti‐miR‐195‐OKSM iPSC colonies. (B) Anti‐miR‐195‐OKSM iPSCs successfully formed EBs *in vitro*. (C) Anti‐miR‐195‐OKSM iPSC lines spontaneously differentiated into 3 germ layers (endoderm, mesoderm, and ectoderm). (D) Anti‐miR‐195‐OKSM iPSCs formed teratomas *in vivo*. Teratoma formation assay demonstrated differentiation of anti‐miR‐195‐OKSM iPSCs into the 3 germ layers as assessed by hematoxylin and eosin staining.

The SiPSCs colonies derived from old SkMs obtained after transduction with OKSM together with miR‐195 inhibitor (miR‐195i‐OKSM‐SiPSCs) were analyzed to investigate further whether miR‐195 inhibition influences pluripotency. Colonies were stained positive for the known pluripotency‐associated markers Oct‐4, SSEA‐1, and Nanog (Fig. 4), and nuclei were stained by DAPI. As SSEA‐1 is one of the earliest markers for mouse iPSCs, some smaller iPSCs cluster on MEFs expressed only SSEA‐1 (Fig. 4D–F). The resultant miR‐195i‐OKSM‐SiPSCs could be induced to form EBs (Fig. 5B) and had no karyotype changes (Fig. 5A). Spontaneous differentiation of these EBs expressed proteins from all three germ layers as assessed by immunocytochemistry (Fig. 5C). Furthermore, miR‐195i‐OKSM‐SiPSCs was injected into athymic nude mice and the formation of subcutaneous teratomas was screened to test their differentiation potential *in vivo*. All three germ layers were found in teratomas derived from miR‐195i‐OKSM‐SiPSCs (Fig. 5D), indicating that miR‐195 inhibition has no effect on pluripotency of iPSCs. We also confirmed that scramble control did not affect pluripotency of iPSCs derived from old SkMs (Fig. S7).

### miR‐195 targets and inhibits Sirt1 in SkMs

**Figure 6:**
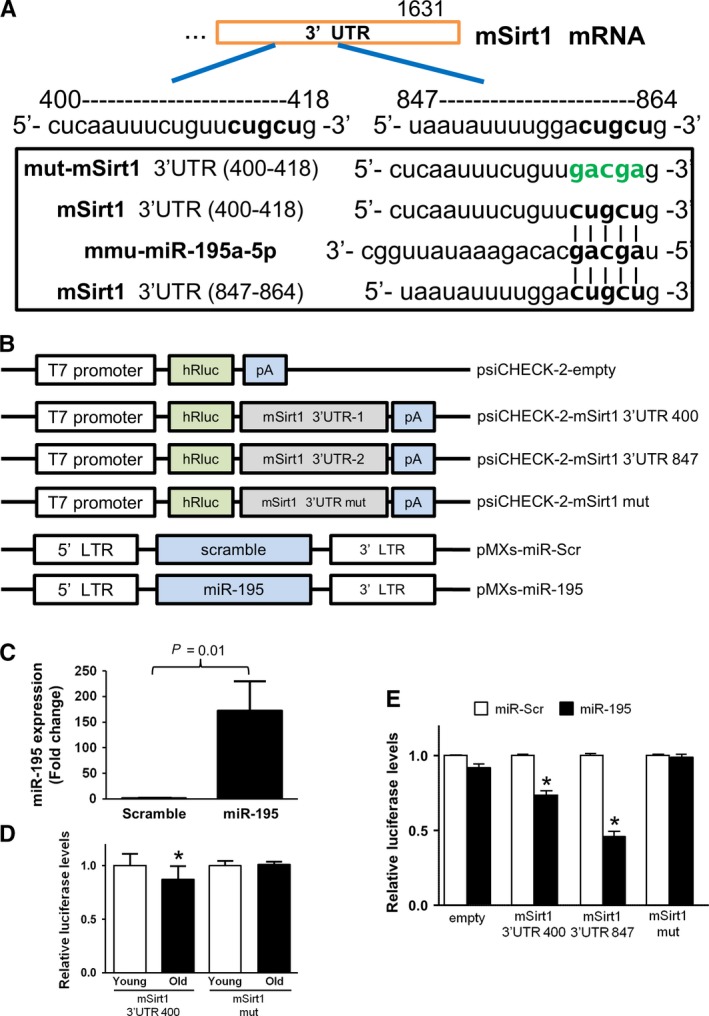
Sirt1 is one of the direct targets of miR‐195. (A) Two putative target sites of miR‐195 in the Sirt1 mRNA 3′‐UTR. miR‐195 was predicted to regulate the Sirt1 expression with complementarities to its seed region that is 2–7 from the miR 5′ end, expecting their strong interaction. (B) Construction map of psiCHECK‐2‐mSirt1 3′ UTR luciferase reporter plasmids and precursor miR‐195 expression clone. (C) qRT–PCR showed successful transfection and significantly higher expression of miR‐195 in young SkMs using pMXs‐miR‐195 plasmid compared with pMXs‐miR‐Scr transfected SkMs. (D) Cotransfection of young SkMs with psiCHECK‐2 vector containing mSirt1 3′ UTR (400–418) or (847–864) together with a plasmid encoding miR‐195 showed decreased luciferase activity (3′ UTR 400: *P* < 0.0001 vs. pMXs‐miR‐Scr transfected cells, 3′ UTR 847: *P* < 0.0001 vs. pMXs‐miR‐Scr transfected cells, respectively). (E) Transfection of old SkMs with psiCHECK‐2 vector containing mSirt1 3′ UTR (400‐418) showed decreased luciferase activity (*P* < 0.005 vs. psiCHECK‐2‐mSirt1 3′ UTR 400 transfected young SkMs).

Three databases were examined (miRANDA, Sanger Mirbase, and Targetscan algorithms) to search for the putative targets of miR‐195 and investigate further the mechanistic participation of miR‐195 in Sirt1 regulation. This resulted in two consensus putative target sites of miR‐195 in the 3′‐UTR of Sirt1 mRNAs (Fig. 6A). Luciferase assay was then performed to confirm the role of Sirt1 as a target gene of miR‐195. A precursor miR‐195 expression vector (pMXs‐miR‐195) was cotransfected with the vectors containing one of the target sites of 3′‐UTR in the Sirt1 gene, because the full length of 3′‐UTR in the Sirt1 gene is too long to insert into a vector. One vector contained 497 bp (32–528 bp) of 3′‐UTR in the Sirt1 gene (psiCHECK‐2‐mSirt1 3′UTR 400) and the other contained 231 bp (744–974 bp) (psiCHECK‐2‐mSirt1 3′UTR 847). A mutant within the Sirt1‐miR‐195 response elements (32–528 bp) in 3′UTR of Sirt1 (psiCHECK‐2‐mSirt1 mut) was generated using site‐directed gene mutagenesis, showing sequences contained 5′‐CUCAAUUUCUGUU***GACGA***G‐3′ (the five bold and italic nucleotides are substitutes for CUGCU) (Fig. 6A–B). qRT–PCR showed successful transfection and significantly higher expression of miR‐195 in cultured young SkMs transfected with pMXs‐miR‐195 compared with those transfected with scramble control vector (pMXs‐miR‐Scr) (Fig. 6C). The cotransfection of pMXs‐miR‐195 with either psiCHECK‐2‐mSirt1 3′UTR 400 or psiCHECK‐2‐mSirt1 3′UTR 847 led to reduced luciferase activity compared with the vector with a scrambled sequence as a negative control (pMXs‐miR‐Scr), indicating forced expression of miR‐195 can downregulate Sirt1 expression via targeting the 2 sites of the 3′‐UTR in this gene. Consistent with these findings, the cotransfection of pMXs‐miR‐195 with psiCHECK‐2‐mSirt1 mut did not reduce luciferase activity in old SkMs, as is the case with pMXs‐miR‐Sc (Fig. 6D). Intriguingly, when we transfected psiCHECK‐2‐mSirt1 3′UTR 847 into either cultured young SkMs or old SkMs, luciferase activity was higher in the young SkMs than in old SkMs, suggesting cultured old SkMs contained much more miR‐195 that interact with psiCHECK‐2‐mSirt1 3′UTR 400 by binding to the target sites (Fig. 6E).

## Discussion

Previous studies have shown that older and senescent cells are more resistant to reprogramming (Li *et al*., 2009; Kim *et al*., 2010; Cheng *et al*., 2011; Wang *et al*., 2011; Rohani *et al*., 2014). These observations have established the importance of current efforts underway to understand the biology of cell reprogramming in the context of chronological and replicative age.

Results of this study demonstrated that the expression of aging markers (such as SA‐β‐gal expression) was increased in old SkMs with shorter telomere length and confirmed that SkMs become resistant to reprogramming as they age. Previous reports demonstrated that age‐dependent decline in reprogramming efficiency is correlated with activated intrinsic senescence pathways in cells from old donors (Zhao *et al*., 2008; Kawamura *et al*., 2009; Li *et al*., 2009). Reprogramming SkMs into iPSCs reverses several cellular and molecular characteristics associated with aging, including senescence, telomere truncation, mitochondrial dysfunction, and global changes in gene expression. This could be of significant interest to elderly people because many age‐associated deficits that were once thought to be permanent may be reversible (Lapasset *et al*., 2011). Interestingly, gene expression profiles of iPSCs derived from old mice and human subjects can also be reset to an embryonic‐like state (Li *et al*., 2009; Lapasset *et al*., 2011; Wang *et al*., 2011). For example, reprogramming can trigger an increase in telomere length in cells of both old mice and humans, reversing the erosion characteristics of aged and senescent cells. However, whether the resulting iPSCs can maintain their telomere length over long‐term passages is still subject to debate (Marion *et al*., 2009; Vaziri *et al*., 2010). The epigenetic state of iPSCs derived from old donors is another aspect of reprogramming‐induced rejuvenation that has not been investigated thoroughly.

The role of miRs in regulating reprogramming of aged cells has provided a better understanding of specific factors that alter aging signaling pathways (Smith‐Vikos & Slack, 2012). Results of this study showed that the expression of miR‐195 was increased in older SkMs but decreased during the induction of *de novo* reprogramming of old SkMs into iPSCs. Although previous studies have demonstrated that miR‐195 is associated with cardiac hypertrophy and heart failure by controlling cell cycle‐related genes that affect aging (Porrello *et al*., 2011), we hypothesized that upregulated miR‐195 is a senescence‐associated miR in reprogramming. Intriguingly, there was an observed discrepancy between miR‐195 and SA‐β‐gal expression in early passaged old SkMs in this study. Biomarkers for the aging phenotype were detected in early passaged old SkMs without SA‐β‐gal, although SA‐β‐gal expression appeared in more than five passages in SkMs of old mice, implying the number of blast cells or stem cells in skeletal muscle of old mice would be lower than in skeletal muscle of young mice, and therefore, the population doubling in each cell of cultured SkMs from old mice should be higher than each cell of cultured SkMs from young mice in the same culturing condition with same passages. The expression of SA‐β‐gal acts as a late maker of cell senescence and requires the accumulation of longevity‐associated proteins and stabilization of the telomere. However, miR‐195 could be an earlier marker for aging phenotype and the high expression of miR‐195 in old SkMs might represent a potentially senescent state or a reversible state with aging phenotype.

Differences in genetic background strongly influence reprogramming efficiency (Schnabel *et al*., 2012) and may supersede the impact of age on the quality of iPSCs. Reprogramming potency also seems to be impaired to a greater extent in bone marrow cells than in dermal fibroblasts due to an increase of cell senescence (Li *et al*., 2009; Cheng *et al*., 2011). Current studies are designed to enhance the reprogramming efficiency of aging somatic cells by targeting the signaling pathways of cell aging. One goal of the present study was to determine how donor age affects reprogramming and whether manipulating of miRs might diminish barriers for reprogramming in aged cells in the mouse model. Results demonstrated that the inhibition of miR‐195 restored the expression of rejuvenation genes (such as SIRT1 and TERT) and increased telomere length. The inhibition of miR‐195 actually facilitated the reprogramming of older SkMs into iPSCs, suggesting a role in which it might act as a negative mediator of somatic reprogramming and could be a potentially useful target to overcome epigenetic barriers related to aging in the derived iPSCs. Our previous report (Ahmed *et al*., 2011) demonstrated that reprogrammed SkMs may have both the ability for cardiac muscle cell differentiation from SiPSCs and a low risk for teratoma formation, and overcoming the epigenetic predisposition to revert to the parent cell type would be a significant improvement for potential clinical applications. Interestingly, our unpublished data indicate that miR‐195 is also upregulated in mouse bone marrow cells. Future studies will be designed to determine whether we can achieve a more efficient reprogramming using the miR‐195 inhibitor with bone marrow cells as a source of iPSCs in aged mice.

Having collected data indicating that reprogramming efficiency can be enhanced by the inhibition of miR‐195, our next goal was to determine whether the pluripotency or quality of derived iPSCs could be influenced by genetic manipulation. Characterization and functional analysis of the derived iPSCs showed that miR‐195 inhibition has no effect on pluripotency. Because the transduced OKSM can sufficiently activate the expression of endogenous pluripotent genes in reprogrammed cells, miR‐195 expression is not a requisite factor for initial reprogramming. In the last phase of reprogramming, the infected cells eventually stabilize into the pluripotent state and most of exogenous viral transduced genes are epigenetically silenced (Stadtfeld *et al*., 2008; Buganim *et al*., 2013). In addition, the pluripotent gene networks are not direct targets of miR‐195 and accordingly are not impacted by the genetic manipulation of miR‐195.

We focused on the potential targets of miR‐195 to determine the underlying mechanisms driving the increase in reprogramming efficiency by miR‐195 inhibition. Bioinformatics prediction and experimental verification demonstrated that miR‐195 can target the age‐associated proteins SIRT1 and TERT. And inhibition of miR‐195 can facilitate reprogramming by reversing their expression in aging SkMs. Evidence already exist showing that SIRT1 is controlled by miR‐195 similar to the findings of this study (Zhu *et al*., 2011). However, we also determined that overexpression of SIRT1 markedly ameliorates OKSM‐induced reprogramming efficiency in old SkMs but not young SkMs. Combining this knowledge with observations that levels of miR‐195 in mesenchymal stem cells are elevated with aging and that the inhibition of miR‐195 reversed the senescent phenotype into juvenile phenotype through a mechanism in which TERT expression was restored (Okada *et al*., unpublished data), this data strongly supports a mechanism in which miR‐195 targets SIRT1 and TERT, or at least an inverse relationship between miR‐195 and SIRT/TERT levels.

The upregulation of SIRT1 by miR‐195 inhibitor might contribute to telomere elongation in iPSCs (De Bonis *et al*., 2014). The original hypothesis that SIRT1 regulates mammalian aging was drawn from two sources. A study of the SIRT1 homolog Sir2 shows that Sir2 prolongs longevity in yeast (Hekimi & Guarente, 2003). Secondly, prolongation of survival by caloric restriction also increases SIRT1 activity (Bordone & Guarente, 2005). Despite these findings about the relationship between SIRT1 and longevity, the role of SIRT1 in the lifespan extension has not been established clearly. For example, moderate expression of SIRT1 attenuates the age‐dependent incidence of cardiac hypertrophy and dysfunction by inducing cardiac resistance to oxidative stress in mice. However, high levels of SIRT1 have been found to increase heart dysfunction (Alcendor *et al*., 2007). In this regard, our previous data showed that depletion of miR‐195 in the heart restored cardiac diastolic function in old mice (Okada *et al*., unpublished data) by putative mechanisms that are guided by SIRT1 when expression levels are restored to similar levels as found in young cells. Therefore, the inhibition of miR‐195 might increase SIRT1 expression levels that would be sufficient to recover the tissue function impaired by aging.

Additionally, the importance of TERT on reprogramming was recently investigated using TERT knockout mice and results showed that the reprogramming efficiency in tail‐tip fibroblasts from TERT‐deficient mice was markedly lower than that from wild‐type mice (Kinoshita *et al*., 2014). TERT is required for elongating telomeres during the reprogramming process, and telomere shortening represents a potent barrier against iPSCs generation in telomerase‐deficient mice (Marion *et al*., 2009). Donor cells that are difficult to be reprogrammed can be more efficiently induced into the pluripotent state by including TERT and SV40 large T antigen with the four Yamanaka factors, highlighting the critical role of TERT in period of iPSC generation (Park *et al*., 2008). Therefore, the restored expression of TERT by miR‐195 inhibitor could facilitate the reprogramming of senescent cells into iPSCs through telomere elongation.

We further interpret the effect of miR‐195 by investigation of downstream genes of the direct targets SIRT1 and TERT. SIRT1 can induce deacetylation of FOXO1, one pluripotent regulator that directly regulates gene expression in either human or mouse ESCs (Zhang *et al*., 2011). Another study showed that knockdown of FOXO1 reduced the reprogramming efficiency as well (Koga *et al*., 2014). Additionally, the age‐dependent decline in reprogramming efficiency could be reversed by silencing p53 expression (Kawamura *et al*., 2009). The present study demonstrated that both protein levels of p53 and FOXO1 dramatically change with age in SkMs and affect reprogramming efficiency. Our findings indicated that the inhibition of miR‐195 restored the expression levels of both SIRT1 and TERT, which in turn resulted in deacetylation of both FOXO1 and p53 in old SkMs as reported previously (Vaziri *et al*., 2001; Motta *et al*., 2004; Xiong *et al*., 2011). Although the acetylation status was not analyzed in the later stage of cultured cells after transduction of miR‐195 inhibitor, we recognize differences between the earlier and the later changes in the old SkMs occurred by the inhibition of miR‐195, and future studies will further reveal the precise mechanisms by which miR‐195 inhibition promotes anti‐aging signaling pathways.

Any given miR may have hundreds of different mRNA targets (Friedman *et al*., 2009). Although this study focuses on SIRT1 and TERT as targets of miR‐195, previous reports on SkMs have identified other targets such as AKT3, ADP ribosylation factor‐like protein 2, B‐cell lymphoma 2 (Bcl‐2), Ccnd, Cdc25, cyclin D1, cyclin E1, and FGF2 (Sekiya *et al*., 2011; Sato *et al*., 2014). It seems logical to assess that miR‐195 may have unexpected effects on other target genes during the reprogramming process. For instance, changes in Bcl‐2 expression by miR‐195 inhibitor might contribute to promote reprogramming in old cells, given that overexpression of Bcl‐2 increases reprogramming efficiency of mouse fibroblasts by two‐ to threefold (Kawamura *et al*., 2009), even though miR‐195 inhibitor (by itself) did not cause karyotypic changes in SiPSCs.

In summary, age‐dependent upregulation of miR‐195 contributes to the barriers for reprogramming old cells into iPSCs. As the growing population of patients with degenerative diseases reach advanced age, inhibition of miR‐195 could be part of a strategy used to improve reprogramming efficiency for autologous cell therapies using skeletal muscle as the starting material for iPSCs.

## Experimental procedures

### Isolation of primary skeletal myoblasts (SkMs)

This study conformed to the Guide for the Care and Use of Laboratory Animals published by the US National Research Council of The National Academies (Eighth Edition, 2011) and followed the protocol approved by the Institutional Animal Care and Use Committee, University of Cincinnati.

SkMs were isolated from C57BL/6 mice (young: 8–10 weeks, The Jackson Laboratory; old: 24–26 months, National Institute on Aging, USA) as previously described (Gharaibeh *et al*., 2008). The isolated cells were then suspended in growth medium (DMEM supplemented with 10% FBS, 10% horse serum, 0.5% chick embryo extract, and 2.5% penicillin/streptomycin) and transferred to coated flasks. After 1 h, the supernatant was withdrawn from the flask and transferred to another coated flask. The cells that adhered rapidly within the first hour of incubation consisted mainly of fibroblasts. The serial transfer of the supernatant with suspended cells was repeated when 30–40% of the cells had adhered to each flask. After five or six serial platings, cultured cells were maintained in FBS‐rich medium (DMEM supplemented with 20% FBS and 2.5% penicillin/streptomycin). Fresh primary cultured cells were used within five passages for all experiments.

### Generation of mouse iPS cells (iPSCs) from SkMs

Reprogramming SkMs derived from young or old C57BL/6 was performed according to previous reports (Takahashi & Yamanaka, 2006). Briefly, retroviral supernatants were harvested from HEK‐293T cell cultures (5 × 10^6^ cells per 100‐mm‐diameter dish) transduced with the ecotropic packaging plasmid pCL‐Eco (4 μg) together with one of the following retrovirus constructs (4 μg): pMXs‐Klf4, pMXs‐Sox2, pMXs‐Oct4, or pMXs‐c‐Myc (obtained from Addgene, Cambridge, MA, USA). Both a lentiviral vector expressing inhibitor for miR‐195 and a scrambled control vector were obtained from the GeneCopoeia^™^ (Rockville, MD, USA). Both carried the mCherry reporter gene. HIV‐based lentiviral vector was transduced into HEK‐293T cells according to the manufacturer's protocol (obtained from GeneCopoeia^™^). Two days after transduction, the supernatant containing viruses was collected and passed through a 0.45‐μm filter. SkMs (1 × 10^5^ cells per well in 6 well, passage 3–5) were infected (day 0) with pMXs‐based retroviruses together with HIV‐based lentivirus for miR inhibition or scrambled control. On day 4, cells (~ 10 000) were passed onto an irradiated mouse embryonic fibroblasts feeder layer in a 6‐cm culture dish. The next day, the medium was replaced with knockout DMEM containing 15% serum replacement (Life Technologies, Carlsbad, CA, USA), nonessential amino acids (Life Technologies), GlutaMAX (Life Technologies), β‐mercaptoethanol, and LIF (1,000 U mL^−1^). Medium was changed every other day, and cellular changes in plates were monitored. After 2–3 weeks, the total number of iPSCs colonies was counted after staining plates for ALP activity following the manufacturer's instructions (Sigma‐Aldrich, St Louis, MO, USA). Based on previously published studies suggesting that exogenous gene silencing might distinguish a pluripotency state, we calculated the number of iPSCs colonies using exogenous mCherry silencing, with morphology resembling mouse ESCs and ALP‐positive staining as the defining criteria (Hotta & Ellis, 2008; Zhao *et al*., 2008). Colonies that satisfied these criteria (except ALP staining) were selected and expanded on feeder fibroblasts using the standard procedure.

### Real‐time PCR

Total RNA including miRs was extracted from cells with miRNeasy micro kit (Qiagen, Hilden, Germany). Total RNA (200 ng) was reverse‐transcribed with Hispec buffer according to the manufacturer's protocol for quantitative RT–PCR (Qiagen). The primer sequences for miR‐195 and miR‐195‐5p inhibitor for quantitative RT–PCR were as follows: miR‐195, forward 5′‐ACACTCCAGCTGGGTAGCAGCACAGAAAT‐3′ and universal 5′‐CCAGTGCAGGGTCCGAGGTA‐3′: miR‐195‐5p inhibitor, forward 5′‐CCCACAACGAGGACTACA‐3′ and reverse 5′‐CGTGAAGAATGTGCGAGAC‐3′. The primer sequences for Sirt1, Tert, and Gapdh gene used for RT–PCR were as follows: Sirt1 gene, forward 5′‐TTGTGAAGCTGTTCGTGGAG‐3′ and reverse 5′‐GGCGTGGAGGTTTTTCAGTA‐3′; Tert gene, forward 5′‐CTGCGTGTGCGTGCTCTGGAC‐3′ and reverse 5′‐CACCTCAGCAAACAGCTTGTTCTC‐3′; and Gapdh gene, forward 5′‐TGGCCTTCCGTGTTCCTACC‐3′ and reverse 5′‐TGTAGGCCATGAGGTCCACCAC‐3′. A pair of PCR primers for U6 and SYBR Green probes was purchased from Qiagen. Reactions were completed in triplicate for each sample and were analyzed using the CFX touch real‐time PCR detection system (Bio‐Rad Laboratories, Hercules, CA USA). Data were normalized to GAPDH or U6 levels. For the detection of miRs, the cDNA for each sample was mixed with a universal primer, a forward primer, and probes obtained from Qiagen.

### Western blot

Cell extracts were prepared using RIPA buffer, resolved on 7.5 or 12.5% polyacrylamide gels, transferred to nitrocellulose, and hybridized using antibodies against acetyl‐FOXO1 (sc‐49437, 1:200; Santa Cruz Biotechnology, Santa Cruz, CA, USA), acetyl‐p53 (#2570, 1:1000; Cell Signaling Technology, Danvers, MA, USA), actin (sc‐1616, 1:3000; Santa Cruz Biotechnology), FOXO1 (#9454, 1:1000; Cell Signaling Technology), Gapdh (G9545, 1:10000; Sigma‐Aldrich), p16 (sc‐1207, 1:200; Santa Cruz Biotechnology), p21 (sc‐6246, 1:50; Santa Cruz Biotechnology), p53 (#2524, 1:200; Cell Signaling Technology), SIRT1 (07–131, 1:200; Millipore Corporation, Billerica, MA, USA), and TERT (ab104588, 1:200; Abcam, Cambridge, UK).

### Embryoid body formation

Mouse iPS cell lines derived using Klf4, Sox2, Oct4, and c‐Myc along with miR‐195 inhibitor (miR‐195i‐iPSCs) were collected by Accutase (Life Technologies) and then resuspended in knockout DMEM supplemented with 15% serum replacement, nonessential amino acids, and GlutaMAX. The cells were plated on 100‐mm prepared adherent dishes and incubated for 40 min to separate the iPSCs from the MEF feeder layer. Collected supernatants were allowed to form embryoid bodies (EBs) in 100‐mm nonadherent dishes for 3 days in a 37°C, 5% CO2 incubator. The formed EBs were then seeded on gelatin‐coated plates, and cultures were maintained for 2 weeks changing the medium every other day.

### Pluripotency markers and embryoid body analysis

The colonies of miR‐195i‐iPSCs plated on cell chamber slides (Thermo Fisher Scientific, Waltham, MA, USA) were fixed in 4% paraformaldehyde at room temperature (RT) for 5 min and then permeabilized with 0.5% Triton for 10 min. After blocking with casein buffer (Thermo Fisher Scientific), cells were incubated with primary antibodies (1:100) used for staining Oct4 (Cell Signaling Technology), SSEA1 (Cell Signaling Technology), and Nanog (Cell Signaling Technology). Alexa Fluor‐conjugated secondary antibodies at a dilution of 1:200 (Thermo Fisher Scientific) were added for 1 h. After staining with DAPI (1:200) to highlight nuclei, immunofluorescence images were taken using fluorescent microscopy.

### Teratoma formation assay

Subconfluent undifferentiated iPSCs were harvested and resuspended in cold HBSS for teratoma formation studies. iPSCs were injected subcutaneously in NU/J mice (The Jackson Laboratory, Bar Harbor, ME, USA) using prechilled syringes with 27‐G needles. Mice were sacrificed for assays 4 weeks after transplantation.

### FISH for telomere length and miR‐195 expression analyses

Telomeres were detected by FISH with peptide nucleic acid telomere Cy3‐labeled probe (PNA Bio, Thousand Oaks, CA, USA) as previously described (Igura *et al*., 2013). Primary cultured cells without passage were prepared for independent experiments each time. Then, these cells were passaged onto cell culture glass slide (Electron Microscope Sciences, Hatfield, PA, USA). The next day, the cells were fixed with 4% paraformaldehyde for 1 h. Fixed cells were incubated with RNase (100 ng μL^−1^) for 20 min and 0.005% pepsin for 5 min at 37 °C. After dehydration by immersing the slides sequentially in 70, 90, and 100% cold ethanol, cells were incubated with 200 nm telomere probe for 10 min at 85 °C and incubated for 2 h at room temperature. Cells were then incubated with 2 ×  SSC for 10 min at 60 °C. DAPI and Cy3 signals were acquired simultaneously into separate channels using a confocal microscope (Zeiss LSM510 META Confocal Microscope, Carl Zeiss AG, Oberkochen, Germany). Telomere length was measured using TFL‐TeloV2‐2 free software (Vancouver, BC, Canada). The integrated fluorescence intensity value for each telomere, which is proportional to the number of hybridized probes, was calculated and presented. TFL‐Telo was used to estimate the length of telomeres from captured images of metaphases that have been stained for Q‐FISH analysis (Poon *et al*., 1999).

To detect miR‐195, primary cultured cells were incubated with 25 nm locked nucleic acid miR‐195 fluorescein amidite‐labeled probe (Exiqon, Denmark) overnight at 37 °C and incubated with 2 ×  SSC for 10 min at 60 °C. DAPI and fluorescein amidite signals were acquired together into separate channels using a fluorescent microscope (Olympus, Tokyo, Japan).

### Senescence‐associated β‐galactosidase staining

SA‐β‐gal is now a widely used biomarker in studies of cellular senescence because it is very easily detected and the activity is strongly associated with cellular senescence, although there is no specific marker to detect cellular senescence (Dimri *et al*., 1995). SA‐β‐gal activity was detected according to the manufacturer's protocol (Bio Vision, Milpitas, CA, USA).

### Luciferase reporter assay

Precursor miR‐195 expression clone was constructed in a vector system (pMXS‐miR‐195) and luciferase reporter vectors (psiCHECK‐2) containing the different two wild‐type sites (32–528, 744–974) of 3′‐UTR of mSirt1 and mutant sequence, respectively. For transfection, SkMs were plated in triplicate into 12‐well plates and cotransfected with 1 μg of pMXs‐miR‐195 (or pMXs‐miR‐Scr) and reporter vector using the Xfect (Clontech Laboratories, Inc., Mountain View, CA, USA). Renilla and Firefly luciferase activities were measured using the Dual Luciferase Reporter Assay System Kit (Promega, Madison, WI, USA), according to the manufacturer's instructions. Transfection efficiency was normalized on the basis of Firefly luciferase activity.

### Statistical analysis

All values were expressed as the mean ± SEM unless otherwise indicated. Significant differences between two groups were determined by unpaired Student's t‐test and among groups of three or more by one‐way analysis of variance (ANOVA). If data did not follow Gaussian distribution, significant differences between the groups were analyzed by the Mann–Whitney U‐test (for two groups) or by the Kruskal–Wallis test (for more than three groups). Differences were considered statistically significant when *P* < 0.05.

## Funding

No funding information provided.

## Conflict of interest

The authors have no conflict of interest to declare.

## Author contributions

HK was involved in study conception and design, data acquisition, analysis, interpretations, and manuscript writing. HWK contributed to study conception and design, data acquisition, analysis, interpretations, and manuscript writing. LW was involved in data acquisition, analysis, and interpretation. MO participated in study conception and data interpretations. CP was involved in review of manuscript. RWM was involved in review of manuscript. YW contributed to study conception and review of manuscript.

## Supporting information


**Fig. S1** mRNA expression of Sirt1 and Tert increases in old SkMs.

**Fig. S2** Protein expression of aging markers increases in old SkMs.

**Fig. S3** Inhibition of age‐induced miR‐195 reverses mRNA expression of Sirt1 and Tert.
**Fig. S4** Inhibition of age‐induced miR‐195 reverses protein expression of SIRT1 and TERT.

**Fig. S5** Expression of miR‐195 is downregulated in reprogrammed iPSCs.
**Fig. S6** Inhibition of age‐induced miR‐195 doesn't affect reprogramming efficiency in young SkMs.

**Fig. S7** Characterization of iPSCs produced from young SkMs with miR‐Scramble (miR‐Scr‐OKSM‐SiPSCs).

**Fig. S8** Inhibition of SIRT1 increases reprogramming efficiency in old SkMs.
