# Supplementary figures and images for: Blockade of senescence‐associated microRNA‐195 in aged skeletal muscle cells facilitates reprogramming to produce induced pluripotent stem cells
Source: Aging Cell. 2015 Dec 5;15(1):56–66. doi: 10.1111/acel.12411 (PMC4717278; doi:10.1111/acel.12411)

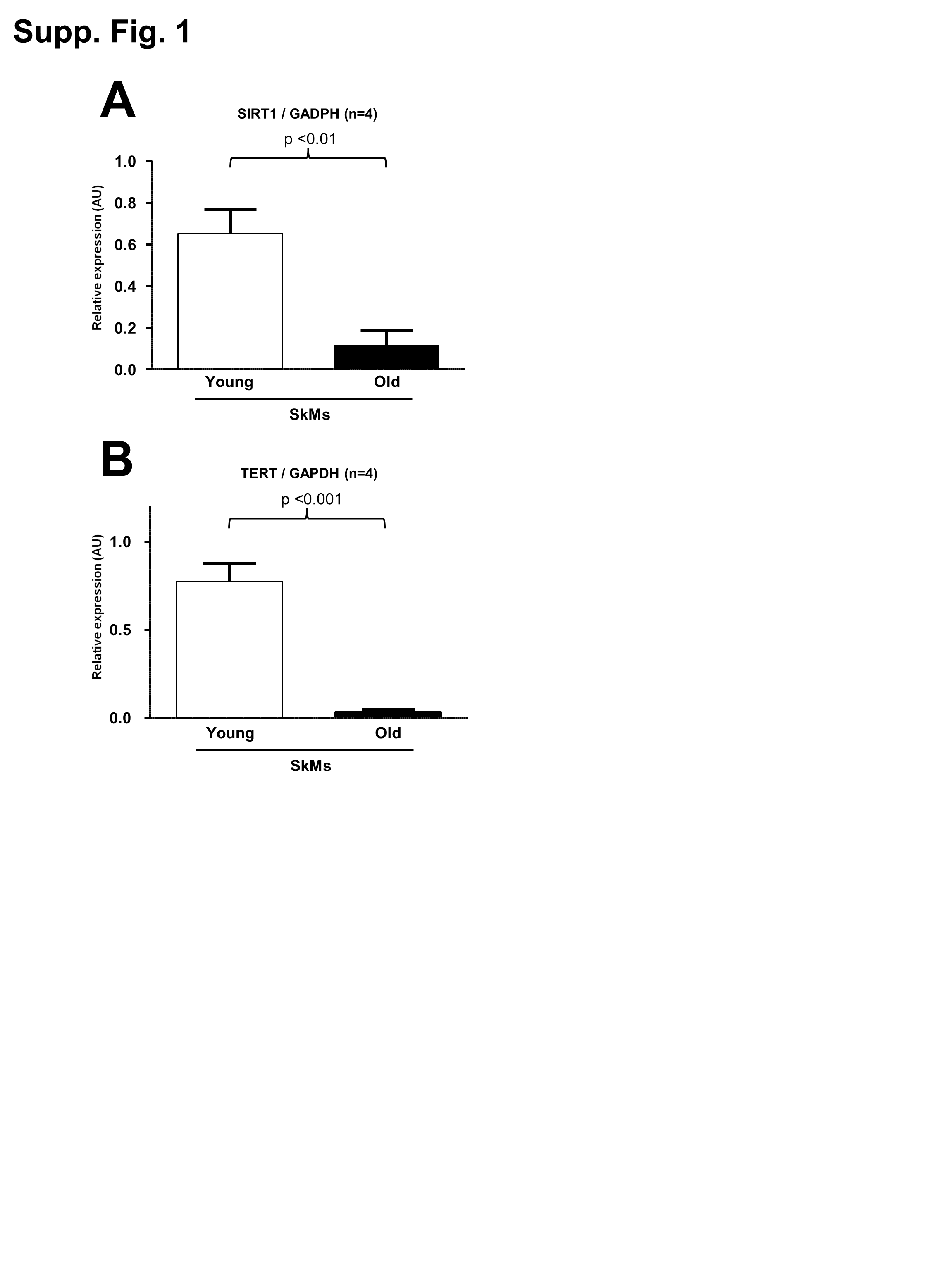

Supplement: Supplementary file 1 — Fig. S1 mRNA expression of Sirt1 and Tert increases in old SkMs. [file ACEL-15-056-s001.tif]

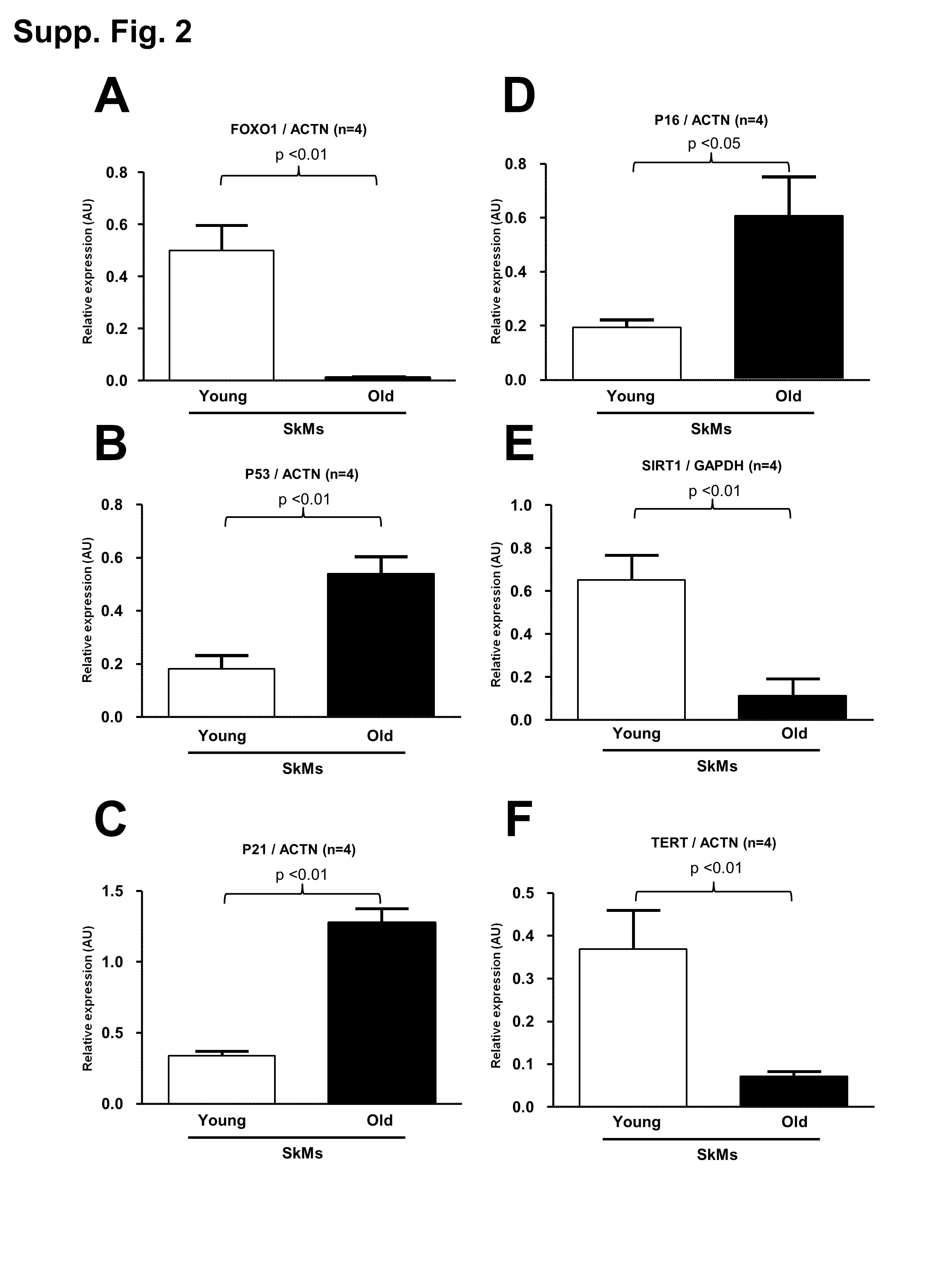

Supplement: Supplementary file 2 — Fig. S2 Protein expression of aging markers increases in old SkMs. [file ACEL-15-056-s002.tif]

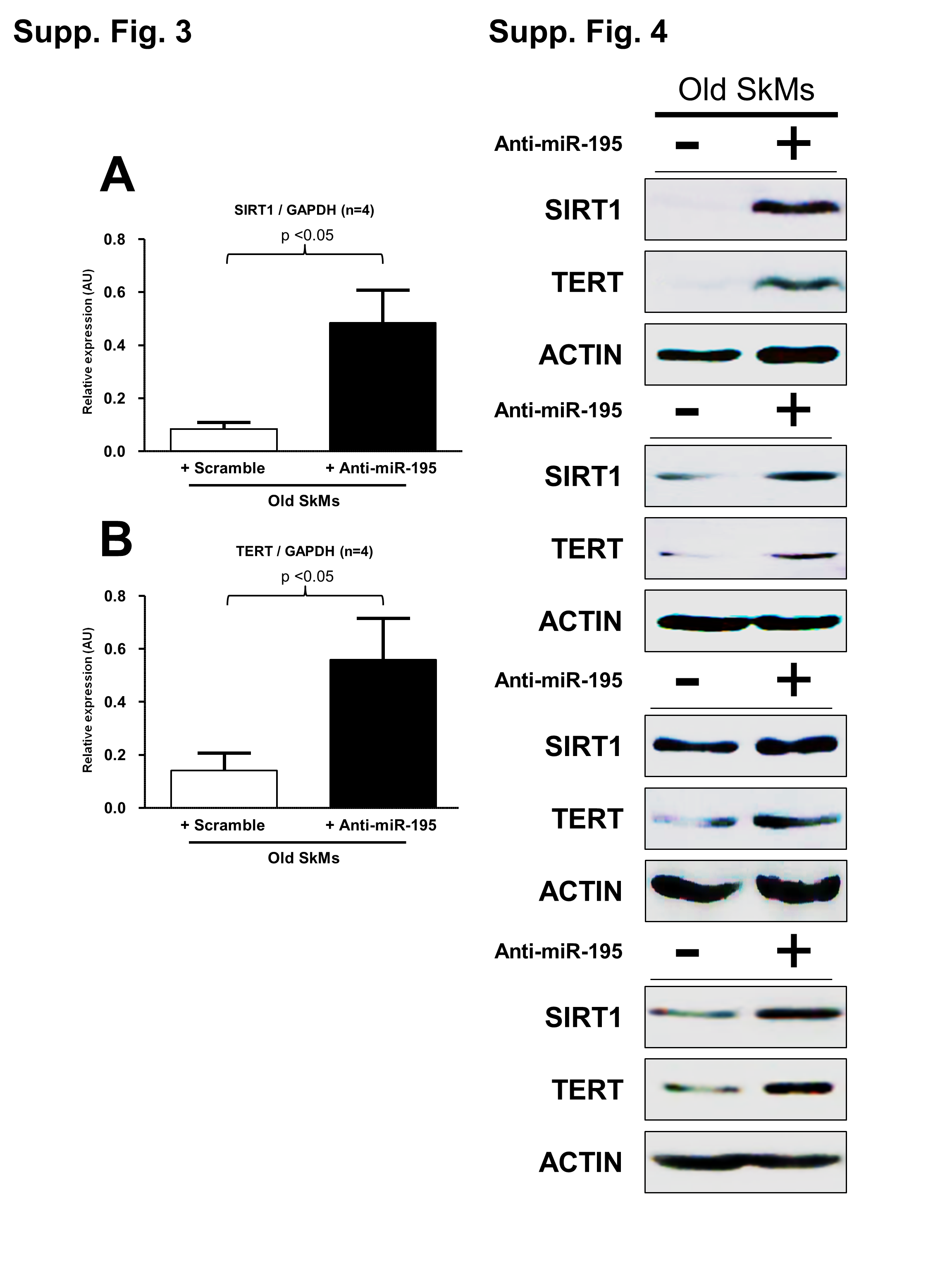

Supplement: Supplementary file 3 — Fig. S3 Inhibition of age‐induced miR‐195 reverses mRNA expression of Sirt1 and Tert. Fig. S4 Inhibition of age‐induced miR‐195 reverses protein expression of SIRT1 and TERT. [file ACEL-15-056-s003.tif]

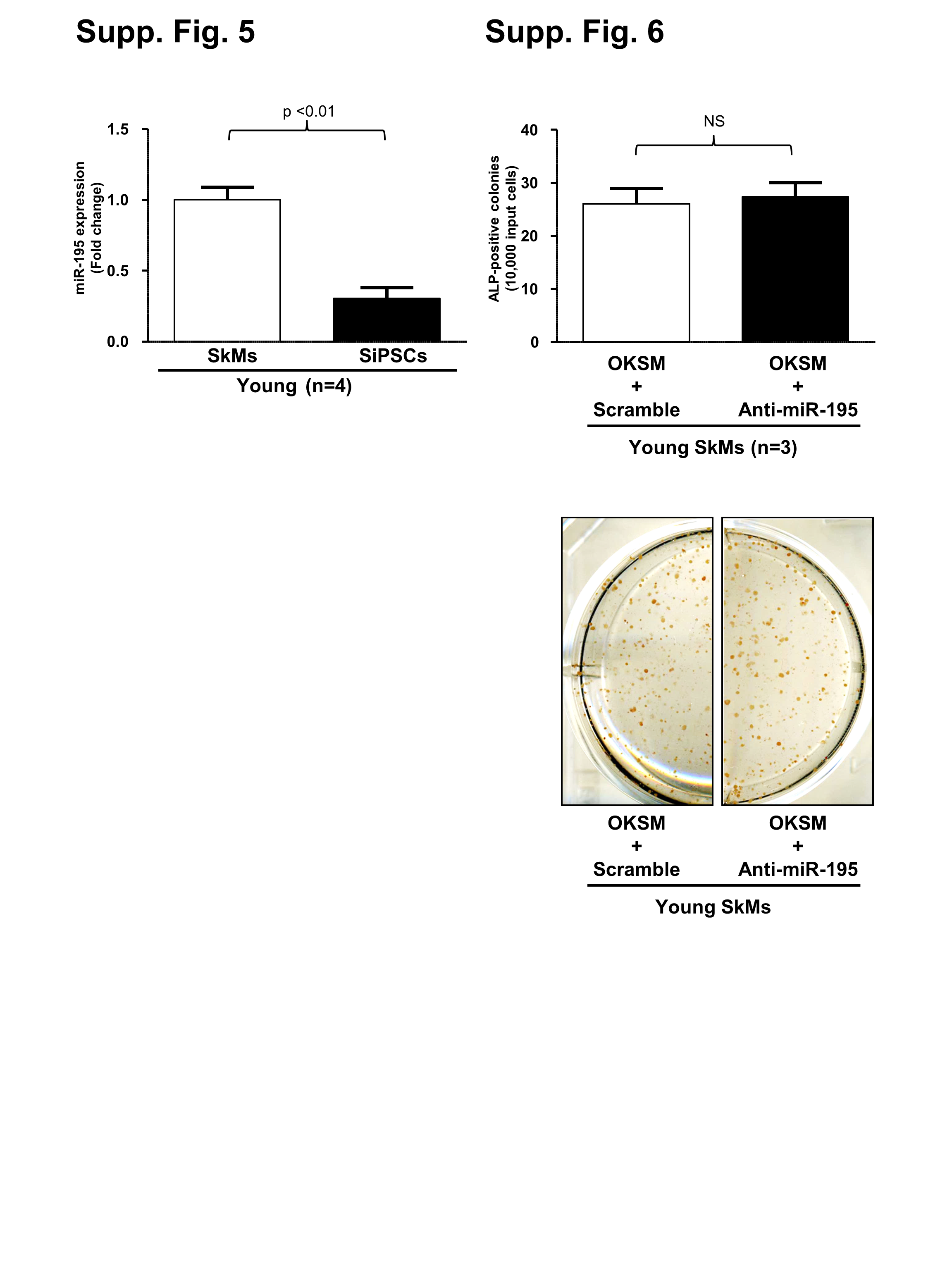

Supplement: Supplementary file 4 — Fig. S5 Expression of miR‐195 is downregulated in reprogrammed iPSCs. Fig. S6 Inhibition of age‐induced miR‐195 doesn't affect reprogramming efficiency in young SkMs. [file ACEL-15-056-s004.tif]

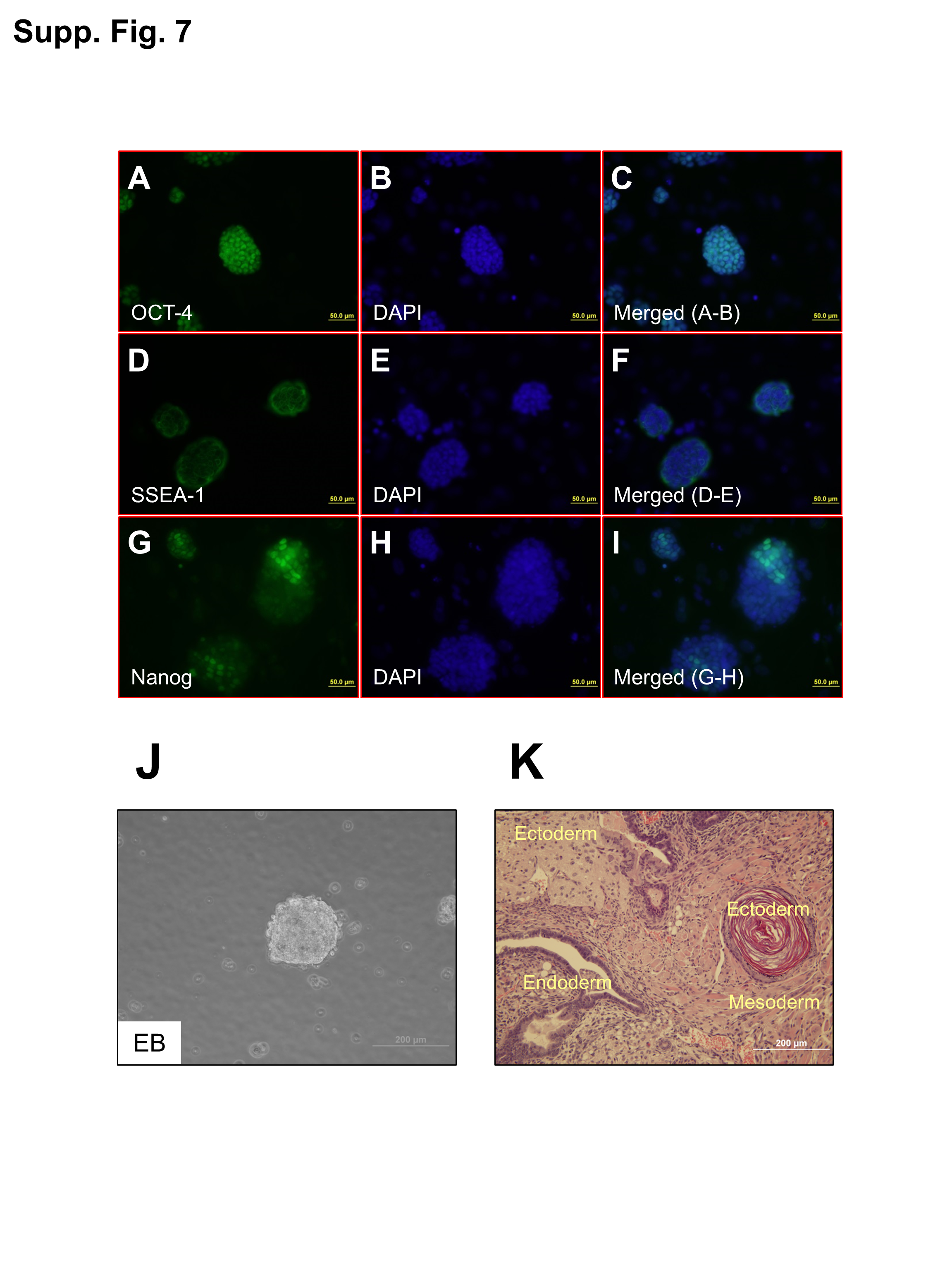

Supplement: Supplementary file 5 — Fig. S7 Characterization of iPSCs produced from young SkMs with miR‐Scramble (miR‐Scr‐OKSM‐SiPSCs). [file ACEL-15-056-s005.tif]

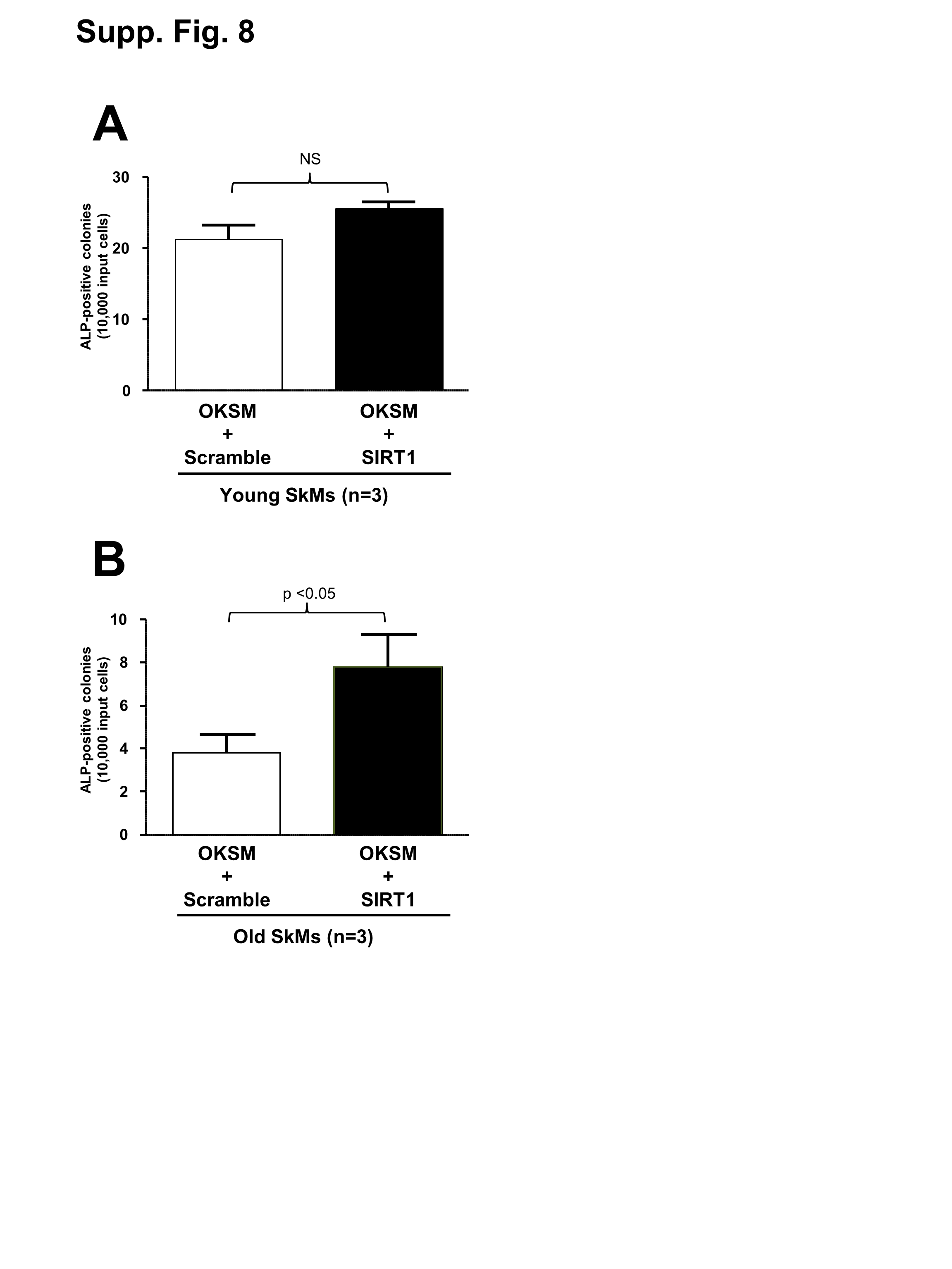

Supplement: Supplementary file 6 — Fig. S8 Inhibition of SIRT1 increases reprogramming efficiency in old SkMs. [file ACEL-15-056-s006.tif]
